# Advances in Pediatric Acute Promyelocytic Leukemia

**DOI:** 10.3390/children7020011

**Published:** 2020-02-02

**Authors:** Shannon E. Conneely, Alexandra M. Stevens

**Affiliations:** Department of Pediatric Hematology/Oncology, Texas Children’s Cancer Center, Baylor College of Medicine, Fannin Street, Houston, TX 77030, USA

**Keywords:** t (15; 17), acute myeloid leukemia, arsenic trioxide, all-trans retinoic acid, outcome, pediatric, PML-RARA, ATRA

## Abstract

Acute promyelocytic leukemia (APL) is a rare disease accounting for only 5%–10% of pediatric acute myeloid leukemia (AML) and fewer than 1000 cases occur annually in the United States across all age groups. Characterized by t (15; 17), with a resultant *PML-RARA* gene fusion driving leukemia development, advances in therapy have improved outcomes for APL significantly in the past several decades, now making APL the most curable form of AML in both children and adults. Cure rates in APL are now comparable to pediatric B-lymphoid leukemias. The success of APL treatment is due, in part, to the breadth of understanding of the driver *PML-RARA* mutation as well as collaborative efforts to quickly introduce and maximize the benefit of new therapies. Here, we review the presentation, clinical features, pathogenesis, and treatment advances in pediatric APL.

## 1. Introduction

Acute promyelocytic leukemia (APL) is a unique entity in acute myeloid malignancies typically characterized by the balanced translocation t (15; 17)(q24.1;q21.2) and resultant *PML-RARA* fusion gene [1,2]. The *PML-RARA* protein product has been identified as the primary driver responsible for nearly all cases of APL, and enhanced understanding of the mechanism by which *PML-RARA* leads to APL has drastically altered the therapeutic approach and outcomes for this disease. Patients with APL now experience the highest cure rates of pediatric acute myeloid malignancies, with an average overall survival (OS) near 95% and event-free survival (EFS) of 90% due to the combined use of all*-trans* retinoic acid (ATRA) and arsenic trioxide (ATO) to induce the terminal differentiation of APL blasts [3,4].

However, some patients with APL still experience complications in this disease and treatment, with early death prior to or shortly after the initiation of therapy accounting for the majority of fatalities, particularly in high-risk patients [5,6,7]. These early deaths are not typically included in EFS and OS rates as many patients die before they can be enrolled on a clinical trial, and, therefore, the actual survival rates of patients diagnosed with APL is lower than reported [8]. Deaths are often due to bleeding complications related to coagulopathy at diagnosis or complications of differentiation syndrome (DS), a unique syndrome in APL caused by excessive numbers of maturing myeloid cells occurring within the first two weeks after the initiation of therapy [5,6,7]. In addition to early deaths, the ongoing use of cytotoxic chemotherapy, such as high dose cytarabine and anthracyclines, further exposes patients to severe treatment side effects such as left ventricular systolic dysfunction and prolonged neutropenia with an increased risk of fatal infections [9]. Current studies are therefore aimed at reducing therapy-related and associated long-term toxicities while maintaining high cure rates, and the early results are promising. The majority of APL clinical trials allow for pediatric patients but primarily include the adult population, and thus data specific to children often lags behind that of adults. Here, we will review the presentation, pathophysiology, and current treatment approaches to pediatric APL.

## 2. Clinical Features

Acute myeloid leukemia (AML) in pediatrics consists of a heterogenous group of diseases previously classified by morphology using the French-American-British (FAB) classification, with the FAB-M3 subtype representing APL. Associations between cytogenetic changes and patient outcomes have since shifted the focus toward cytogenetic classification to distinguish between types of AML and allow for risk-stratified therapy. APL, characterized by t(15;17)(q24.1;q21.2), accounts for only 5%–10% of pediatric AML and increases in prevalence with age [10]. It is found in less than 2% of infants with AML and subsequently increases steadily through adolescence and young adulthood, with a peak incidence in the 4^th^ decade of life [10]. APL occurs equally in males and females among all age groups [9,11]. Risk factors associated with APL development have primarily been investigated in adults. Case reports of therapy-related APL following etoposide for other cancers have been reported, and one recent study suggests that obesity increases the risk for APL [12,13,14]. To date, no pediatric-specific risk factors have been identified.

While APL is considered a favorable cytogenetic feature in risk stratification of AML as a whole, additional risk groups within APL have been defined, allowing for risk-adapted therapy. A white blood cell (WBC) count at diagnosis has proved to be the most effective predictor of outcome, and patients presenting with a WBC less than 10,000 cells/μl are considered to be at standard risk (SR) of relapse whereas those presenting with a WBC greater than or equal to 10,000 cells/μl are categorized as being at high risk (HR) of relapse [15]. HR patients therefore receive more intensive upfront therapy to mitigate relapse risk, but they also suffer from higher rates of early death due to coagulopathy and complications of therapy [8,11].

A unique presenting feature of APL is profound coagulopathy comparable to disseminated intravascular coagulation (DIC). Baseline coagulation tests should be routinely assessed in all patients with leukemia at initial presentation, as the presence of a profound coagulopathy may be an early indicator of APL. The severity of coagulopathy varies from isolated laboratory abnormalities to life-threatening bleeding episodes. In addition to thrombocytopenia, patients with APL may have low fibrinogen, prolonged prothrombin and activated partial thromboplastin times, and elevated D-dimer [5,16]. The coagulopathy is primarily due to the altered expression of various coagulation factors in the APL blasts themselves. The blasts demonstrate an increased expression of tissue factor, cysteine protease, Annexin 2, tissue plasminogen activator, and urokinase-like plasminogen activator receptor which cause both a hypercoagulable state and increased fibrinolysis [17,18]. In addition, increased elastase plus the depletion of α2 antiplasmin and a decreased thrombin activatable fibrinolysis inhibitor further increase fibrinolysis [19]. The end result is an increased bleeding risk, which must be closely monitored and aggressively treated with transfusion support. Serious bleeding events have recently been reported in roughly 15% of pediatric APL patients with up to 10% of children suffering fatal bleeding injuries in some series [5,6,8,20]. Death due to intracranial or pulmonary hemorrhage is the leading cause of early death in APL and is seen primarily in HR patients [5,8]. While advances in therapy have significantly reduced the risk of relapse in APL, rates of early death due to bleeding events have unfortunately remained the same [21].

The diagnosis of APL is reliant on the identification of t (15; 17) and *PML-RARA* in blast cells, yet cytogenetic results are often not immediately available and thus a high index of suspicion for APL must exist in order to initiate therapy quickly and reduce the risk of immediate morbidity or mortality from bleeding episodes. Coagulopathy at diagnosis should raise clinical suspicion, as should the presence of certain features of the leukemia cells themselves. Auer rods, linear azurophilic granules within the cytoplasm of blast cells composed of myeloperoxidase, are common in APL, though they may also be seen in other AML subtypes [22]. Immunophenotyping of APL cells typically demonstrates CD13, CD33, CD117, and myeloperoxidase positivity with a high side-scatter [23]. In addition, immunophenotyping may reveal absent or low expression of CD34, HLA-DR, CD10, CD11a, CD11b, CD117, and CD18, with low level HLA-DR, CD11a, and CD18 having the most diagnostic power. CD56 is a unique marker found in 10% of APL cases and has been suggested as a poor prognostic indicator, with a higher risk of relapse in adult patients [24]. The combinations of these clinical and cellular features may raise suspicion of APL enough to initiate therapy prior to confirmation of the *PML-RARA* transcript.

Unlike other types of AML where extramedullary involvement of leukemia is common, patients with APL rarely demonstrate disease outside of the bone marrow. APL blasts in the cerebrospinal fluid (CSF) at presentation or relapse were previously described only in case reports, though CSF is not routinely tested at diagnosis [25,26]. Recent data from the Children’s Oncology Group showed that up to 25% of children had demonstrable APL blasts in the CSF in the absence of a traumatic tap when CSF was tested [9]. However, patients with CSF involvement at diagnosis do not appear to have an increased risk of relapse unless intracranial hemorrhage occurs, and therefore only patients with intracranial hemorrhage are currently recommended to receive CNS-directed therapy [20]. Diagnostic lumbar punctures are now avoided in APL as they introduce an added bleeding risk without therapeutic benefit. Other extramedullary presentations of APL, such as myeloid sarcoma and chloromas, are also rare but have been noted in case reports, primarily at relapse [27,28,29]. 

A final feature unique to APL is the risk of differentiation syndrome (DS) following the initiation of treatment with ATRA and ATO, which occurs in up to 20% of children [9,30]. These medicines induce the differentiation of APL blasts to mature myeloid cells, and when this occurs in excess, DS can result, leading to life-threatening complications. Patients develop weight gain, fever, respiratory distress, hypotension, and even renal failure due to excessive numbers of maturing myeloid cells leading to endothelial damage and edema [31]. Patients with HR disease are at greater risk of DS, though it may occur in SR patients with a low presenting WBC count as well [9]. The early recognition and treatment of DS is necessary to minimize fatalities, and patients should be closely monitored for DS after the initiation of therapy [31]. Treatment strategies include steroids and hydroxyurea as soon as DS is suspected, though leukapheresis should be avoided as it does not affect outcomes and subjects patients to unnecessary bleeding risk [31,32,33,34]. As DS is a significant contributor to early death in APL, recent studies have employed prophylactic steroids to prevent DS with successful reductions in DS-related deaths [11]. Patients presenting with severe illness due to presumed APL should be started on therapy with ATRA plus steroids with or without hydroxyurea immediately even before *PML-RARA* transcripts can be confirmed, as delaying therapy may actually worsen DS in such patients, and early treatment does not typically preclude patients from enrolling on clinical trials. 

## 3. Pathophysiology

The balanced translocation t (15; 17)(q24.1;q21.2) is the cytogenetic hallmark of APL and the most common mutation driving APL development, described in 95% of APL cases [9,11,35]. The translocation joins the Promyelocytic leukemia (*PML*) gene on chromosome 15 with the retinoic acid receptor alpha (*RARA*) gene on chromosome 17, leading to the *PML-RARA* fusion gene and the PML-RARα protein [36,37,38]. The mechanism by which PML-RARα leads to APL development has been well-described over the past several decades. PML-RARα retains the ability of RARα to bind retinoic acid responsive elements and dimerize with retinoid X receptor protein but inhibits normal gene transcription regulated by these elements, ultimately leading to the repression of RARα target genes and blockade in differentiation at the promyelocyte stage [37]. An additional mechanism of disease initiation was more recently described wherein PML-RARα prevents the formation of PML nuclear bodies (NBs) [39]. NBs typically lead to the activation of p53 tumor suppressor pathways and induce cellular senescence under stress situations [37]. Therefore, PML-RARα also leads to the failed activation of p53 with decreased cell death and increased self-renewal. Together, the failure of myeloid differentiation with uncontrolled proliferation contribute together to the development of leukemia.

While the classic translocation described above is by far the most common lesion producing the *PML-RARA* fusion, cryptic rearrangements or complex cytogenetic changes can also occur. Cryptic rearrangements lead to the production of *PML-RARA* transcripts in the absence of definitive t (15; 17) cytogenetics via fluorescent in situ hybridization and conventional karyotype analysis. Such cases are uncommon but have been described previously in case reports [40]. Therefore, the presence of *PML-RARA* transcripts is a more specific diagnostic tool than t (15;17) alone [36,41]. Even within t (15; 17) (q24.1; q21.2), there is variability in *PML-RARA* owed to variations in the *PML* gene breakpoint. The *RARA* breakpoint is almost always in intron 2 of the *RARA* gene, however, the *PML* breakpoint can occur in one of 3 different clusters, termed bcr [42]. Bcr1 occurs in intron 6 and produces a long isoform mRNA. Bcr2 occurs in exon 6 and produces variable isoform mRNA. Bcr3 occurs in intron 3 and produces short isoform mRNA. The prevalence of each bcr varies with ethnicity and geographic location, with bcr1 most common overall followed by bcr3 then bcr2 [43]. Pediatric data are limited but show similar rates of bcr subtypes as adults with variations related to ethnicity [43,44]. Adult studies have suggested that bcr may have prognostic significance, with reports of higher relapse rates in bcr2 and higher presenting WBC and associated poorer prognosis in bcr3 [45]. To date, there has not been enough evidence to incorporate bcr into risk stratification for adults or children. 

When APL is diagnosed in the absence of t (15; 17) and *PML-*RARA transcripts, an atypical *RARA* fusion partner can often be identified. In these cases, the breakpoint of *RARA* is similar to that seen in *PML-RARA*. Additional fusion partners include *ZBTB16, NPM1, STAT5B, BCOR, PRKAR1A, FIP1L1,* and *NABP1* [46,47,48,49]. Cases with these atypical *RARA* fusion partners often vary from traditional APL as many lack Auer rods on morphologic evaluation, have an average lower age at presentation, and may be less responsive to differentiation therapy [44,46]. There are also cases of APL that lack *RARA* rearrangement altogether, but instead demonstrate fusions with other members of the retinoid signaling family [44]. Most APL clinical trials exclude patients with atypical or absent *RARA* rearrangements, and therefore the outcomes of these patients are limited to small case series and case reports. One series reported that 9 of 18 patients with atypical APL were transitioned onto AML treatment regimens, which showed superior EFS compared to APL therapy [44].

In addition to *RARA* rearrangements, co-occurring mutations and cytogenetic changes in APL have been identified in a majority of children. Mutations in FMS-like tyrosine kinase gene (*FLT3*) are the most common, described in up to 40% of pediatric cases [44,50]. There are 2 primary *FLT3* mutations that occur, internal tandem duplications (*FLT3*-ITD) and a missense mutation at amino acid 835, both leading to constitutive activity of the tyrosine kinase receptor [44,51,52]. Both of these mutations are also described in other types of AML and generally confer a poorer prognosis [53]. The use of *FLT3* mutations as a prognostic indicator in APL has remained controversial and is not currently factored into risk stratification [45,50,54]. Studies in both children and adults have shown that patients with *FLT3* mutations have a higher WBC at presentation and are at a higher risk of death [6,50,55]. Results published by the Children’s Oncology Group in 2012 demonstrated a median presenting WBC of 32.95 in patients with *FLT3* mutations compared to a median of 3.6 in patients with wild-type FLT3, and 70% of patients with a *FLT3* mutation had a presenting WBC above 10,000 whereas only 26% of patients with wild-type *FLT3* fell into the same category [50]. In addition, induction deaths occurred exclusively in children with *FLT3* mutations and 40% of patients failed to achieve complete remission (CR) following induction, though OS and EFS were similar between groups [50]. It is important to note that treatment regimens have changed since these data were published with continued improvements in CR, EFS, and OS amongst all patients, and thus these same results may not be demonstrated in current trials. 

## 4. Treatment Advances

Prior to the characterization of APL as the unique disease we know it as today (with a defining genetic mutation), APL was treated with conventional chemotherapy (cytarabine and daunorubicin) similar to other types of AML. Cure rates were poor, with remission rates less than 60% and high rates of induction death due to bleeding and infectious complications [56]. Following the discovery of *PML-RARA*, the use of differentiation therapy with ATRA has significantly improved outcomes and become the standard of care (Table 1). ATRA binds to PML-RARα, inducing a conformational change leading to the recruitment of a proteasome and subsequent degradation of the fusion protein [37,39]. This allows wild-type RARα to resume normal function, thereby driving the differentiation of the promyelocytic leukemia cell to a mature myeloid cell.

When ATRA was introduced over 3 decades ago, it was first trialed as a single agent and provided CR rates of 75%–85% in adult studies [57,58]. However, relapses remained common and thus combination therapy with both ATRA and conventional chemotherapy became standard. Using concurrent therapy, patients of all ages began to experience relapse rates of less than 10% at 2 years [33,59]. Numerous studies have attempted to optimize chemotherapy regimens and identify the most essential agents to improve outcomes. Anthracyclines appeared to be the most important chemotherapeutic agent to decrease relapse rates, though the exact anthracycline used in treatment plans may vary. Unfortunately, the total dose of anthracycline used in most regimens is very high and associated with short- and long-term cardiotoxicity [60]. The contribution of cytarabine to improving outcomes has remained controversial, as some studies show superior outcomes with cytarabine in induction whereas others do not [61,62]. Cytarabine does increase the risk of prolonged myelosuppression and subsequent infectious complications. Current studies aim to further reduce chemotherapy exposure, and risk-adapted therapy has recently allowed for the removal of traditional chemotherapy for SR patients and decreased cumulative anthracycline doses for HR patients [4].

The use of ATO has added to treatment success in APL and has further challenged the need for conventional chemotherapy for patients with APL. ATO was introduced in the 1990s primarily as a relapse therapy given as a single agent [63]. ATO triggers apoptosis in APL cells and maturation in promyelocytes, working synergistically with ATRA to induce differentiation [37]. Following the success of ATO in relapsed patients, it was then trialed in front-line therapy in combination with ATRA, though it was initially limited to post-induction courses. In children, these studies yielded an average OS of 94% and EFS of 91% and demonstrated noninferiority to traditional chemotherapy plus ATRA even though cumulative anthracycline doses were lower than those used in the historical controls [9]. The APML4 trial by the Australasian Leukaemia and Lymphoma Group (ALL-G) was the first large clinical trial to include ATO in induction therapy in combination with ATRA and included both adult and pediatric patients [11]. This study concurrently removed anthracyclines from all SR therapy and limited anthracycline in HR therapy to induction alone, with the complete removal of cytarabine from all treatment groups. Even with significant reductions in chemotherapy exposure, the combined use of ATO and ATRA in induction and post-induction therapy achieved EFS and OS of 92% and 96%, respectively, for SR patients and 83% and 87% for HR patients with a cumulative relapse risk of 5% in all groups [4]. The combination of ATO and ATRA without chemotherapy is now the standard of care for SR adults, though HR patients still receive anthracyclines during induction. Recent trials exclusively in pediatric patients aim to emulate these outcomes in children with risk-adapted and response-based therapy to minimize the use of chemotherapy, but data are currently maturing and have not yet been published. Thus, the current standard of care for children with APL remains ATRA plus ATO in combination with chemotherapy for all patients. 

Prolonged maintenance cycles with oral chemotherapy regimens have also long been part of APL treatment. Whether or not maintenance therapy is required to maintain current outcomes is also debated. A Cochrane review analyzed data from ten different trials and over 2000 patients of varying ages with or without the use of maintenance therapy [64]. Treatment regimens varied widely between studies though, as some used ATRA alone, some used chemotherapy alone, and some used combined therapy. The data showed no significant improvement in OS with or without maintenance therapy or between different types of maintenance therapy regimens, though disease-free survival was improved with any type of maintenance therapy. However, the use of ATO in front-line treatment has further challenged the benefit of maintenance therapy, as recent results from the Italian-German APL0406 study demonstrated a 5-year OS of 99.2%, an EFS of 97%, and a relapse rate of less than 2% in SR adults treated with ATRA and ATO alone [65,66]. Studies in HR patients have not been published. The most recent Children’s Oncology Group APL study, AAML1331, removed maintenance therapy from both SR and HR treatment groups to validate these results in children.

The advancements demonstrated in APL therapy have caused a shift in focus toward minimizing unnecessary therapy and improving the tolerance to treatment. The primary aims of most current trials consist of maintaining high CR, EFS, and OS while limiting exposure to chemotherapy and making treatment more tolerable with an improved quality of life. ATO in the United States is only approved in intravenous formulations, therefore requiring prolonged hospital admissions or daily home or clinic infusions in order to receive therapy. Recently, ATO has been developed in an oral formulation with high bioavailability and similar effectiveness to the intravenous form in international studies [67,68]. In addition, oral ATO has fewer QTc prolonging side effects than the intravenous form, further reducing potential side effects of therapy. Studies utilizing oral ATO in pediatric patients are occurring at international sites to determine if children achieve the same responses as adults, as pharmacologic properties of medications can vary with age.

The addition of ATRA and ATO to upfront therapy for APL has resulted in resistance to these treatments in the few patients who do relapse [69]. Resistance to ATRA is primarily due to mutations of the binding domain of RARα, demonstrated in 40% of relapsed adults previously exposed to ATRA therapy, thereby reducing the binding affinity of ATRA in these cases. The mechanism of resistance to ATO therapy is not well described. Current relapse therapies include gemtuzumab ozogamicin (GO), an antibody-drug conjugate targeting CD33 expressing cells, or clinical trials utilizing tamibarotene, a synthetic retinoid with higher binding affinity for PML- RARα. The former is approved for 2-year-old children and older in relapsed APL expressing CD33, whereas tamibarotene is still under investigation. Small molecule inhibitors with targets such as tyrosine kinases, telomerase, and c-myc have demonstrated some efficacy in preclinical studies using APL cell lines but have not yet advanced to clinical trials [70,71,72,73].

## 5. Conclusion

Due to the characterization of the *PML-RARA* gene caused by t (15; 17) and its effect on leukemia development, patients with APL now benefit from a targeted therapeutic approach primarily utilizing differentiation therapy. This has allowed for significant advances in OS and EFS, making APL the most curable form of AML today. With combined ATRA and ATO therapy, children and adults with HR disease experience an OS of greater than 85% with less than a 5% risk of relapse at 5 years. Outcomes in SR patients are even better with OS near 95% and a relapse risk of less than 5%. The primary risk of death in HR patients occurs early in therapy due to coagulopathy at presentation and complications of differentiation syndrome, though strategies to predict and mitigate these risks are being investigated. 

The focus of APL therapy has now shifted to minimize exposure to chemotherapeutic agents and associated side effects with promising results. Adult studies have successfully reduced anthracycline exposure while maintaining high cure rates, and similar pediatric studies are ongoing. New challenges in the future will include developing effective relapse regimens, as patients exposed to ATO and ATRA in initial chemotherapy are often resistant at relapse, though luckily the number of patients who relapse is small. The identification of rare *RARA* fusion partners causing atypical APL also poses unique challenges, as these patients have variable responsiveness to current differentiation therapy.

While outcomes in other pediatric AML types have remained unchanged for many years, the story of APL provides hope that better outcomes are possible. Beyond APL, differentiation therapy has since been investigated in other pediatric cancers, including use in maintenance therapy for neuroblastoma. The success of APL now serves as a paradigm for targeted therapy in cancer treatment with revolutionary advances in outcomes, which we hope to emulate in other cancers moving forward. 

## Figures and Tables

**Table 1:** Summary of the drugs used in acute promyelocytic leukemia (APL) therapy.

Drug	Mechanism of Action	Role in Current Therapy
ATRA	Binding and degradation of PML- RARα fusion protein	Standard treatment including induction and post-induction
ATO	Induces apoptosis of APL cells	Standard treatment including induction and post-induction
Anthracyclines	Inhibition of DNA synthesis	Only used in induction for HR patients
Tamibarotene	Synthetic retinoid with mechanism similar to ATRA	Clinical trials for relapsed disease
Gemtuzumab Ozogamicin	Induces cell death of CD33 expressing cells via anti-CD33 drug conjugate	Approved for relapse therapy
